# Outbreak of monophasic Salmonella Typhimurium Sequence Type 34 linked to chocolate products

**DOI:** 10.1016/j.amsu.2022.104597

**Published:** 2022-09-08

**Authors:** Sejal Lund, Maliha Tahir, Laiba Imran Vohra, Amatul Hadi Hamdana, Shahzaib Ahmad

**Affiliations:** a Shaheed Mohtarma Benazir Bhutto Medical College, Karachi, Pakistan; b Mayo Hospital, Lahore, Pakistan; c Ziauddin University, Karachi, Pakistan; d Dow Medical College, Karachi, Pakistan

**Keywords:** Monophasic salmonella typhimurium, Outbreak, Antimicrobial resistance, Chocolate products, Salmonellosis, Foodborne illness, Salmonella enterica serovar 1,4,[5],12:i:

## Abstract

As of 3rd June 2022, 445 cases of monophasic Salmonella Typhimurium sequence type 34 infection had been reported globally. The outbreak was caused by two novel strains of monophasic S. Typhimurium with unusual multi-drug resistance. The majority of these cases involved children aged 10 or younger, and they had a hospitalization rate higher than most previous outbreaks of monophasic S. Typhimurium, but no fatalities were recorded. The infection was traced to certain Belgian chocolate products after extensive microbiological and epidemiological research. Public health officials took immediate action to recall all the contaminated products, and the risk of exposure was reduced. The common symptoms are bloody diarrhea, acute onset of fever, abdominal pain, and vomiting. This article aims to thoroughly review the recent outbreak of monophasic Salmonella Typhimurium ST-34, including its epidemiology and comparison with ongoing outbreaks. We also highlighted past chocolate-related salmonella outbreaks and current control and prevention guidelines and recommendations.

## Highlights

• The present salmonellosis outbreak in Europe was traced to certain chocolate products produced at a plant in Belgium.
• The majority of these cases involved children aged 10 or younger.
• This outbreak was caused by two novel strains of monophasic S. Typhimurium with unusual multi-drug resistance.
• Rare drug resistance to aminoglycosides, phenicols, and trimethoprim can be used for screening of probable cases.
• For improving food safety, active monitoring, surveillance, inspection, outbreak management, research, and education are needed.

## 1 Introduction

In February 2022, a cluster of monophasic Salmonella Typhimurium sequence type 34 infection cases was reported in the United Kingdom [1]. The outbreak was caused by novel strains of monophasic S. Typhimurium that have unusual multi-drug resistance to six types of antibiotics, namely penicillins, aminoglycosides, phenicols, sulfonamides, trimethoprim, and tetracyclines [2]. According to European Centre for Disease Prevention and Control (ECDC), as of 3rd June 2022, 392 cases had been reported in 12 European Union (EU) and European Economic Area (EEA) countries and the UK. The vast majority of the reported cases were children 10 years or younger, and they had a higher hospitalization rate (approximately 40%) than most of the previous outbreaks of monophasic S. Typhimurium infections [1,3]. Salmonella is a genus of rod-shaped gram-negative bacteria belonging to the family Enterobacteriaceae. It commonly causes salmonellosis, which is one of the most frequently reported foodborne gastrointestinal tract infections characterized by the acute onset of fever, abdominal pain, vomiting, and diarrhea (the current outbreak reported the occurrence of usually bloody diarrhea) [2,4]. The monophasic variant of Salmonella typhimurium, also called Salmonella enterica subsp. enterica serovar 1,4 [5],12:i:- (antigenic formula), has been one of the top three serotypes of salmonella causing human infections in Europe since 2014 [5]. Previously, the carrier foods for salmonellosis usually included eggs and pig meat and their products, as well as bakery products, but the source of recent infections was found to be chocolate products (specifically Kinder products) manufactured at the Ferrero Corporate Plant in Arlon, Belgium between December 2021 and January 2022 [2,4]. The purpose of this article is to provide a comprehensive review of the recent outbreak of monophasic Salmonella Typhimurium sequence type 34, including an analysis of its epidemiology and comparisons to other ongoing outbreaks. Additionally, we have discussed previous salmonella outbreaks linked to chocolate, as well as current control and prevention guidelines and recommendations.

## 2 HOW salmonella gets in chocolate?

According to recent studies, salmonella strains were recorded in the processing equipment for the buttermilk ingredients [6]. Salmonella has always been a huge concern for the cocoa-chocolate factories, and according to the studies, contamination of chocolate with Salmonella has remarkably been associated with the use of cocoa-contaminated ingredients [7]. All the food products grown in the ground have a higher liability of getting contaminated, likewise, cocoa is too at a higher risk of coming in contact with Salmonella and E.coli bacteria as a consequence of contamination by animal waste, improper storage, use of unclean water used during crop production, harvesting, and processing [8]. Furthermore, the high fat content in cocoa seeds seems to protect the Salmonella bacteria from heat and increase its longevity [7,8]. Additional steps involved in the preparation of cocoa-like fermentation, drying, roasting, and storage appear to be the main gateway for the entrance of Salmonella bacteria into the chocolate manufacturing chain [7].

## 3 Past chocolate related salmonella outbreaks

Several episodes of salmonellosis due to salmonella-contaminated chocolate products have been reported previously, as shown in table no. 1 [[9], [10], [11], [12], [13], [14], [15]]. From 2006 to January 2022, no such outbreaks were reported from the European region.

**Table 1:** Past chocolate related salmonella outbreaks.

YEAR	AREAS AFFECTED	NUMBER OF PEOPLE AFFECTED	SOURCE OF INFECTION
**1970**	Sweden	110	Cocoa powder containing confectionary products
**1973**–**1974**	Canada	95	Christmas-wrapped chocolate balls
**1973**–**1974**	USA	30	Christmas-wrapped chocolate balls
**1982**–**1983**	UK	245	Two types of chocolate products produced in Italy
**1985**–**1986**	Canada and US	33	Chocolate coins imported from Belgium
**1987**	Norway and Finland	350	Chocolate
**2001**–**2002**	Germany	439	A specific brand of chocolate supplied extensively through a single supermarket chain
**2006**	UK	56	Chocolate

South Korea reported an outbreak of gastroenteritis in school children in 2018 due to salmonella infected chocolate cake [16].

## 4 Salmonella outbreak 2022

**Fig. 1:**
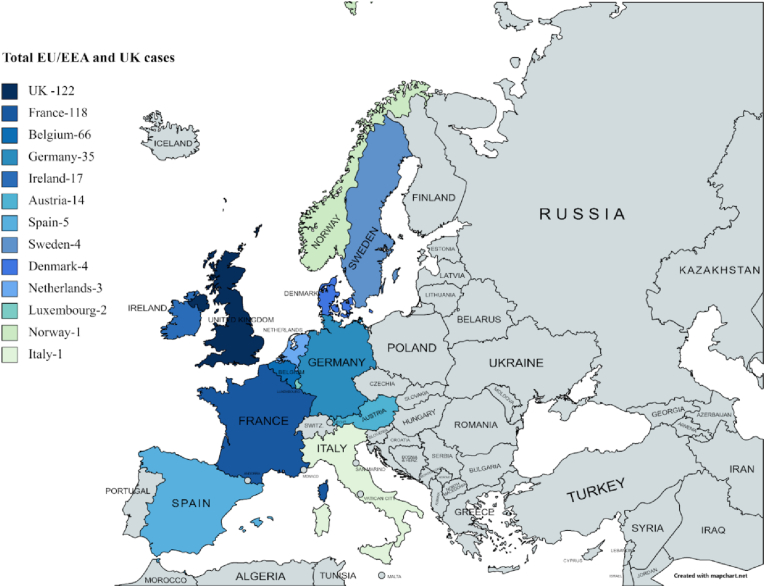
Total case distribution in EU/EEA and UK [1].

According to the European Centre for Disease Prevention and Control (ECDC), as of 3rd June 2022, 392 cases of monophasic Salmonella Typhimurium sequence type 34 infection have been identified in the EU/EEA and the UK. Fig. 1 shows the total case distribution in EU/EEA and the UK.Globally, 445 cases linked to this outbreak have been reported [3]. The cases consist of two novel strains of monophasic S. Typhimurium ST34 that are diagnosed and divided into Cluster 1 and Cluster 2 as per the European outbreak case definitions [17]. These novel strains have shown resistance to more antimicrobials than the previous strains of monophasic S. Typhimurium. Cluster 1 is resistant to about six classes of antimicrobials, i.e., penicillins, aminoglycosides (streptomycin, spectinomycin, kanamycin, and gentamicin), phenicols, sulfonamides, trimethoprim, and tetracyclines. It is of note that the lnu(F) gene, which encodes for resistance to lincosamides, was also present in some strains in Cluster 1. Cluster 2 was found to be resistant to only 4 of these antibiotics, i.e., penicillins/beta lactams, tetracyclines, sulphonamides, and aminoglycosides (streptomycin and kanamycin) [1,17]. Although these strains of monophasic S. typhimurium ST34 are susceptible to amikacin, azithromycin, ciprofloxacin, meropenem, and third generation cephalosporins cefotaxime and ceftazidime, the antimicrobials are not recommended for most mild and moderate cases in healthy people to prevent the development of new resistant strains that could render the medication useless. Most cases are self-limiting, and the severe ones are treated with electrolyte replacement and rehydration [2,17]. The source of infection was found to be chocolate products from Belgium, which were distributed to over 113 countries [2]. The cases in the EU/EEA and the UK ranged in age from 8 months to 56 years, but the vast majority of infections (86.3%) were identified in children aged 10 or younger and females accounted for more reported cases (63.3%) than males, the female-to-male ratio being 1.7. Although no deaths were reported during the outbreak, 41.3% of the cases were hospitalized [1,17].

Epidemiological investigations carried out by Europe's health agency suggested that reported cases were linked to the consumption of the kinder products manufactured by Ferrero (a multinational Italian company that manufactures branded chocolate and confectionery items). Further investigations by Belgium's food safety authority confirmed the presence of monophasic S. Typhimurium, matching the human outbreak cases, in buttermilk tanks at the Ferrero Corporate plant in Arlon, Belgium in December 2021 and January 2022. On 8 April 2022, the Arlon plant in Belgium was suspended following the inspection, and all of its kinder products were recalled, lowering the risk of exposure [2,17,18]. Of note, eight of the reported cases of S. Typhimurium were not linked with chocolate products manufactured at the Arlon plant in Belgium, suggesting either a different source of infection or a secondary infection [17]. Advanced molecular typing techniques were used to identify the affected cases during this outbreak [3]. The resistance to aminoglycosides, phenicols, and trimethoprim is rare in monophasic S. Typhimurium and could therefore be used for screening of probable cases [17].

## 5 Salmonella and comparison with other ongoing outbreaks

Amidst an ongoing pandemic of COVID-19 and an outbreak of acute hepatitis of unknown origin in children, a recent outburst of Salmonella Typhimurium Sequence Type 34 linked to chocolate products has triggered a new health alert. A difference between the symptoms and age distribution of these three diseases is outlined in table no. 2.

**Table 2:** Symptoms and age DISTRIBUTION of salmonellosis caused by salmonella typhimurium sequence type 34, COVID-19, and acute hepatitis in children [2,3,[19], [20], [21], [22], [23], [24], [25]].

DISEASE	SALMONELLOSIS CAUSED BY SALMONELLA TYPHIMURIUM SEQUENCE TYPE 34S	COVID-19	ACUTE HEPATITIS OF UNKNOWN ORIGIN IN CHILDREN
**Symptoms**	Bloody diarrhea (57%), fever, stomach cramps, nausea, vomiting, chills, and headache.	Flu-like symptoms, loss of taste or smell (68%), cough (50%), fever (43%), myalgias (36%), headache (34%), diarrhea (13%), and vomiting (10%).	Jaundice (68.8%), vomiting (57.6%), lethargy (48.6%), diarrhea (43.1%), dark urine, light-colored stools (42.7%), fever (28.5%), and abdominal pain (26.1%)
**Incubation Period**	6 h to 6 days after consumption of the contaminated products.	2–14 days after exposure.	14–28 days after exposure.
**Age distribution**	Most of the cases were among children aged ≤10 years.	All ages.	Children between the ages of 1 month and 16 years.
**Transmission**	Specific chocolate products from a Belgian chocolate factory have been identified as likely vehicles of infection.	Through animal-to-human transmission and human-to-human transmission via respiratory droplets, through contact with an infected person.	Undetermined.
**Management**	Electrolyte replacement, rehydration, Antibiotics, and Intravenous IV fluids.	Antipyretics, analgesics, or antitussives for fever, headache, myalgias and cough respectively, antivirals for mild to moderate symptoms, anti-SARS-CoV-2 monoclonal antibodies for high-risk patients, Invasive mechanical ventilation for respiratory failure	Treatment is usually supportive care.
**Prevention**	Avoiding consumption of implicated chocolate products until allowed by public health authorities, washing hands, washing vegetables, and fruits before consumption.	Frequent hand hygiene, respiratory etiquette, disinfecting surfaces, maintaining physical distance, and wearing masks.	Frequent hand hygiene, avoiding crowded places, good ventilation, wearing masks, safe food handling, regular cleaning of surfaces, and using safe water.

## 6 Challenges

In this outbreak, a high hospitalization rate (about 40%) has been observed. Since advanced molecular typing techniques were used to identify the affected cases and not all countries frequently use this testing approach, some cases may have gone undetected [3]. The products in question were sold in Europe and all around the world, raising the likelihood of additional cases being reported from other countries outside Europe [2]. The scale of the outbreak is undoubtedly underestimated due to the known under-reporting of Salmonella surveillance systems and the different sensitivity levels of microbiological techniques used across nations [1].

According to the first joint update on the salmonella outbreak published on 18 May 2022 by the European Centre for Disease Prevention and Control (ECDC) and European Food Safety Authority (EFSA), there are eight cases that did not report the consumption of Ferrero chocolate products manufactured at the Arlon, Belgium plant but rather the consumption of other Ferrero products. It has not yet been possible to identify the manufacturing plants of those Ferrero products [17].

## 7 Current efforts

After several Salmonella Typhimurium cases were reported in children due to chocolate consumption, risk management actions were carried out in all the affected countries [1]. On 10 April 2022, the World Health Organization/Food and Agriculture Organization International Food Safety Authorities Network (INFOSAN) and the European Rapid Alert System for Food and Feed (RASFF) issued a global health alert. The World Health Organization (WHO) urges all the member states to update if any other potential contaminated product is identified and to report for any recent cases linked with this outbreak [1,2]. The WHO recommended public health guidelines are to be followed, including handwashing with soap after coming in contact with any contaminated item, eating properly cooked food, using boiled milk, and other safety measures to be taken at all stages of the food chain [2]. Furthermore, the European Center for Disease Prevention and Control (ECDC) also encourages the public health authorities to comply with the food safety authorities [19]. Although chocolate contamination is difficult to catch, and a high fat content of chocolate has a protective effect on the bacteria, but still following the above mentioned countermeasures has resulted in decline in the number of cases reported [1,3].

## 8 Recommendations

To prevent further salmonella outbreaks, control measures must be implemented at all phases of the food chain, from agricultural production through food processing, manufacture, and preparation in both commercial and domestic settings [2]. The adoption of an effective quality assurance system during chocolate manufacturing would help to produce safe chocolate, and the system, such as Hazard Analysis and Critical Control Points (HACCP), should be implemented by the chocolate industry [26]. Raw materials should preferably be purchased on demand from reliable suppliers who are aware of the Salmonella concern and have a strict in-house surveillance mechanism in place [27]. Basic personal hygiene requirements should be scrupulously followed by all workers, visitors, and contractors (see Fig. 1). Hand washing and drying, the removal of jewelry, the prevention of hand-to-mouth contact and the provision of proper work gear are all required, to prevent contamination [28]. Since water condensation from cold water pipes, refrigerator coils, and cooling tunnels can readily become Salmonella infection sites, it is important to maintain a dry working environment [27]. Additionally, the eradication of Salmonella requires the roasting of beans or nibs. As a result, it's critical to apply suitable controls throughout the roasting stage to guarantee that adequate temperatures and roasting periods are attained and that no under-roasted material enters the next process [28]. According to the WHO global strategy for food safety 2022–2030 draft, the food safety systems must be transformed from reactive to proactive systems, especially when addressing health risks emerging at the human-animal-ecosystems environment interface, and should be more cost-effective for both importing and exporting countries while enhancing food safety in the domestic market, as it is a shared responsibility [29]. Regulatory frameworks on food safety are necessary to define what is acceptable, establish measures to monitor compliance and address non-compliance, thus protecting the public from unsafe or fraudulent practices [29]. Successfully ensuring food safety from farm to fork requires a more inclusive approach with all stakeholders, including empowered consumers and food business operators [29]. According to WHO's introductory module on strengthening surveillance and response to foodborne diseases, countries may improve their ongoing surveillance and response activities for foodborne illnesses and incorporate them into an already-existing system of national surveillance and response. This will enable them to evaluate the level of their surveillance and response systems in connection to foodborne illnesses, as well as lessen the negative effects on public health, disease burden, and the economy. The framework for strengthening surveillance of and response to foodborne diseases is illustrated in figure no. 2 [30].

**Fig. 2:**
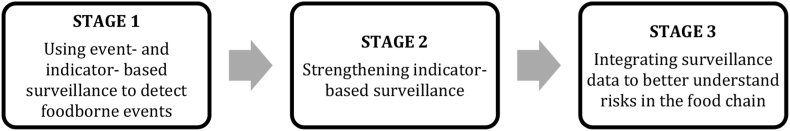
The framework for strengthening surveillance of and response to foodborne diseases..

## 9 Conclusions

In conclusion, this comprehensive review on the outbreak of salmonellosis linked to consumption of chocolate contaminated with monophasic Salmonella Typhimurium ST34 points out that the occurrence of such outbreaks has been implicated in different countries in children in past years. In order to improve food safety in the long run, food safety authorities must become more conscious of the necessity to focus on key functions of safety: monitoring, surveillance, inspection, enforcement, outbreak management, research, and education. The global scope of the outbreak demonstrates how simple it is to spread a tainted item over several nations. This emphasizes the necessity of systems for global surveillance and information sharing to ensure that global epidemics can be dealt with quickly and effectively.

## Sources of funding

No source of funding

## Ethical approval

Not applicable.

## Consent

Not Applicable.

## Author contribution

All authors contributed to every stage of article writing equally, including study concept, design, paper writing, and critical review, and final approval of the version to be published.

## Guarantor

The Guarantor is the one or more people who accept full responsibility for the work and/or the conduct of the study, had access to the data, and controlled the decision to publish.

Corresponding author (Dr. Maliha Tahir)

## Contributions

All the authors contributed equally and substantially in all the steps involved in writing this article.

## Funding

None.

## Declaration of competing interest

No Conflict of interest.
